# The Response of Mitochondrial Respiration and Quantity in Skeletal Muscle and Adipose Tissue to Exercise in Humans with Prediabetes

**DOI:** 10.3390/cells10113013

**Published:** 2021-11-04

**Authors:** Lukasz Szczerbinski, Mark Alan Taylor, Urszula Puchta, Paulina Konopka, Adam Paszko, Anna Citko, Karol Szczerbinski, Joanna Goscik, Maria Gorska, Steen Larsen, Adam Kretowski

**Affiliations:** 1 Department of Endocrinology, Diabetology and Internal Medicine, Medical University of Bialystok, Sklodowskiej-Curie 24A, 15-276 Bialystok, Poland; puchta.urszula@gmail.com (U.P.); szczerbinskikarol@gmail.com (K.S.); mgorska25@wp.pl (M.G.); adam.kretowski@umb.edu.pl (A.K.); 2 Clinical Research Centre, Medical University of Bialystok, Sklodowskiej-Curie 24A, 15-276 Bialystok, Poland; mark.taylor@ucsf.edu (M.A.T.); paulina.konopka@umb.edu.pl (P.K.); adam.paszko@umb.edu.pl (A.P.); anna.citko@umb.edu.pl (A.C.); joanna.goscik@umb.edu.pl (J.G.); stelar@sund.ku.dk (S.L.); 3 Helen Diller Family Comprehensive Cancer Center, University of California at San Francisco, 1450 3rd St., San Francisco, CA 94158, USA; 4 Department of Biomedical Sciences, University of Copenhagen, Blegdamsvej 3, 2200 Copenhagen, Denmark

**Keywords:** type 2 diabetes, obesity, mitochondria, muscle, adipose tissue, fat, exercise, prediabetes

## Abstract

Background: Mitochondrial dysfunction has been implicated in the pathogenesis of type 2 diabetes, but its contribution to the early stages of dysglycemia remains poorly understood. By collecting a high-resolution stage-based spectrum of dysglycemia, our study fills this gap by evaluating derangement in both the function and quantity of mitochondria. We sampled mitochondria in skeletal muscle and subcutaneous adipose tissues of subjects with progressive advancement of dysglycemia under a three-month exercise intervention. Methods: We measured clinical metabolic parameters and gathered skeletal muscle and adipose tissue biopsies before and after the three-month exercise intervention. We then assayed the number of mitochondria via citrate synthase (CS) activity and functional parameters with high-resolution respirometry. Results: In muscle, there were no differences in mitochondrial quantity or function at baseline between normoglycemics and prediabetics. However, the intervention caused improvement in CS activity, implying an increase in mitochondrial quantity. By contrast in adipose tissue, baseline differences in CS activity were present, with the lowest CS activity coincident with impaired fasting glucose and impaired glucose tolerance (IFG + IGT). Finally, CS activity, but few of the functional metrics, improved under the intervention. Conclusions: We show that in prediabetes, no differences in the function or amount of mitochondria (measured by CS activity) in skeletal muscle are apparent, but in adipose tissue of subjects with IFG + IGT, a significantly reduced activity of CS was observed. Finally, metabolic improvements under the exercise correlate to improvements in the amount, rather than function, of mitochondria in both tissues.

## 1. Introduction

Mitochondria play a central role in cellular metabolism. They are the primary source of ATP, the energy currency of the cell, which is primarily produced by the oxygen-dependent process of oxidative phosphorylation. Dysfunction of these organelles has been extensively documented in the tissues of subjects with metabolic disturbances, including insulin resistance and type 2 diabetes (T2D). Specifically, it was shown that patients with T2D have impaired activity of mitochondria in skeletal muscle, liver, fat tissue and even the brain [1]. However, whether this is the result of impaired functioning of these organelles [2,3,4] or the result of a reduction in the number of mitochondria in metabolically active tissues [5] remains unclear. Interest in the role of mitochondria in T2D pathogenesis has increased substantially in the past several years as a result of landmark studies pointing to mitochondria’s lynchpin role in metabolic dysregulation. Several studies showed that insulin-resistant patients have decreased skeletal muscle mitochondrial content [6,7,8,9] and reduced oxidative capacity [4,5,10] with impaired insulin-stimulated ATP production [11,12]. However, Larsen et al. showed no differences in respiratory capacity per mitochondrion, nor the amount of mitochondria in skeletal muscle between subjects with and without T2D matched for activity level and VO_2max_ [13].

Ectopic lipid accumulation in tissues like the liver, adipose tissue and skeletal muscle has been related to a “substrate overload” of mitochondria and impairment of fatty acids oxidation, exacerbating insulin resistance [14]. This likely leads to the common clinical presentation of T2D patients with so-called “metabolic inflexibility” [15]. Mitochondrial metabolic flexibility is the ability of mitochondria to adapt to changes in substrate availability and energy demands, switching between glucose and FFAs as energetic substrates. In T2D, the ability to accomplish this switch from fat to carbohydrates in response to changes in nutrient availability is impaired, leading to the accumulation of intramyocellular lipids and insulin resistance [16,17]. However, previous studies have shown that after an exercise intervention, metabolic flexibility in type 2 diabetic subjects can be restored [18]. It is still debated whether T2D patients’ mitochondrial metabolic inflexibility and its changes under the exercise training are a result of differences in the amount or function of mitochondria [19]. This debate has emerged from the limited availability of evidence of mitochondria function and amount during dysglycemic states preceding overt T2D. Prediabetes also called intermediate hyperglycemia, is just such a preceding state and is a condition that highly predisposes individuals to T2D development [20]. Typically, it is defined as a blood glucose concentration higher than normal healthy, but below the threshold for a diabetes diagnosis: according to the American Diabetes Association (ADA) [21], it is diagnosed when one of the following exists: impaired fasting glucose (IFG) or impaired glucose tolerance (IGT).

A handful of clinical trials focusing on lifestyle intervention have shown that exercise is extremely effective in improving glucose tolerance and preventing the progression of IGT to overt T2D [22,23,24]. Combined metanalyses on T2D prevention in high-risk populations with lifestyle modifications (exercise and diet) have shown a reduction of 30% to 60% in progression to diabetes over a 3- to 5-year time frame with an average risk ratio of T2D development equal 0.59 compared with control [22,23,24]. The mechanism underlying these changes was driven by changes in mitochondrial adaptations in both skeletal muscle and adipose tissue [18,25,26] in both insulin-resistant and healthy subjects. However, there are only a handful of studies investigating the role of mitochondria in the beneficial molecular adaptations after exercise in a prediabetic population [27,28,29].

Mitochondrial adaptations in the development of diabetes and response to exercise can be defined as changes in mitochondrial content [30,31], as well as alterations in mitochondrial respiratory function [18,32,33]. Transmission electron microscopy (TEM) is routinely used to measure mitochondrial volume density (MitoVD) and is the gold standard technique for the assessment of mitochondrial content. However, due to its methodological complexity, it is not scalable; so maximal citrate synthase (CS) activity is widely used as a biomarker of mitochondrial content. Larsen et al. [34] showed that CS activity highly correlates with mitochondrial content measured via microscopy. Several methods for the assessment of mitochondrial respiration exist, including the measurement of mitochondrial ATP production rate. However, the high-resolution respirometry in permeabilized muscle fibers is currently considered the gold standard method for the assessment of mitochondrial respiration [35].

Although concomitant changes in both mitochondrial content and respiration are usually described in various studies, a dissociation between these two has also been observed [35,36,37,38,39]. A better understanding of these in the pathogenesis of diabetes, and how these adaptations occur in the response to exercise in sedentary patients with prediabetes, is crucial to better personalization of exercise prescription aiming at modulating both mitochondrial respiration and amount.

Based on the limited number of studies on the role of mitochondrial dysfunction in the early stages of diabetes development in humans, and unclear evidence of the involvement of mitochondrial respiration and content in the beneficial therapeutic effects of exercise interventions, we aimed to: understand differences in parameters of oxidative phosphorylation and the amount of mitochondria in muscle and adipose tissue, and investigate the effectiveness of exercise intervention to improve metabolic health and its links with changes in mitochondrial parameters.

## 2. Materials and Methods

### 2.1. Studied Population and Study Design

**Figure 1:**
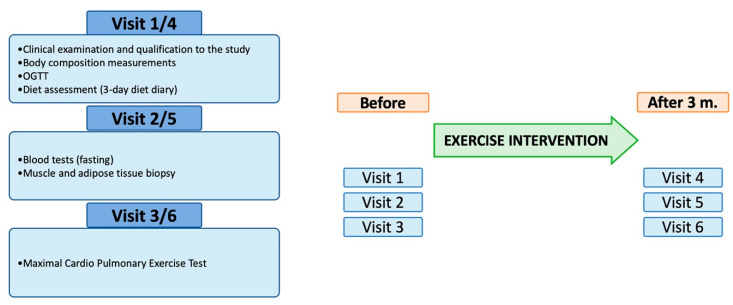
Schematic diagram of the project design and description of each patients’ visit to the study center. OGTT—oral glucose tolerance test.

The study cohort for this project was selected from participants in the exercise intervention study “Bialystok Exercise Study in Diabetes” (BESD), conducted by the Department of Endocrinology, Diabetology and Internal Medicine and Clinical Research Centre of the Medical University of Bialystok. In this project, sedentary males at different stages of dysglycemia living in the city of Bialystok participated in three months of an exercise intervention consisting of supervised training sessions at a local fitness center. All participants underwent clinical assessment at recruitment to the project, followed by oral glucose tolerance test (OGTT), skeletal muscle and adipose tissue biopsy, and maximal cardiopulmonary exercise test (CPET). All tests were performed before the intervention and repeated 3 months following each participants’ final exercise session. A schematic diagram of the BESD project design and description of each patients’ visit to the center are presented in Figure 1. Inclusion and exclusion criteria accompany in Table 1.

For this study, we selected 54 non-diabetic subjects from our panel who had complete before- and after-intervention mitochondrial respiration measurements. They were divided according to ADA and Polish Diabetes Association dysglycemia diagnostic criteria into:

- NG group (*n* = 19)—subjects with normal fasting glucose and normal glucose tolerance, defined as fasting plasma glucose (FPG) <100 mg/dL and 2-h glucose during OGTT (2h-GLU) <140 mg/dL.
- IFG group (*n* = 27)—subjects with impaired fasting glucose and normal glucose tolerance, defined as FPG 100-125 mg/dL and 2h-GLU <140 mg/dL.
- IFG + IGT group (*n* = 8)—subjects with impaired fasting glucose and impaired glucose tolerance, defined as FPG 100–125 mg/dL and 2h-GLU 140–199 mg/dL.

Subjects within these groups were matched for age, body mass index (BMI), the number of performed training sessions during the intervention, and changes in daily kcal consumption. Moreover, the fraction of patients on angiotensin-converting-enzyme inhibitors treatment for hypertension did not differ between groups.

The study was conducted in accordance with the ethical standards of the institutional research committee and with the 1964 Helsinki declaration and its later amendments and was approved by the local ethics committee of the Medical University of Bialystok (approval number: R-I-002/469/2014). All study participants provided written informed consent.

### 2.2. Exercise Intervention

All patients underwent a 3-month exercise intervention composed of mixed training, aerobic and strength exercises, which had previously been shown to be the most effective for diabetes prevention [41,42,43]. The frequency of training was three sessions per week for 12 weeks, for a total number of 36 trainings. For this particular study, only subjects who performed at least 80% of all planned training were included. Each training lasted approximately 85 min, beginning with a warm-up (15 min), strength exercises (40 min), followed by endurance exercises (30 min). For endurance exercise (biking and running on stationary equipment) the intensity was personalized for each patient by adjusting exercise equipment to produce 60–70% of the individual’s VO_2max_. For resistance exercises, loads were 60–75% of 1 Repetition Max for each exercise, ascertained before the intervention and then increased gradually with an individual adjustment every three weeks. Strength exercises involved all main groups of muscles. Exercise sessions were divided into three types, each one involving different muscle groups. During each session, participants performed six types of strength exercises, each in three sets with 10–15 repetitions. Every session type was performed once weekly so that each of the three training sessions per week was different and affected different groups of muscles. The most significant feature of the exercise intervention is that it was 100% supervised to ensure uniformity among patients in the exercise intervention. The myWellness system (Technogym, Cesena, Italy) recorded all the training sessions for every patient including intensity and loads of each exercise. Thus, every training session was carefully measured to ensure that confounding training variation was eliminated.

### 2.3. Muscle and Fat Biopsy

Biopsies of muscle and adipose tissue were performed before and after 3 months of the intervention. They were performed between 7:30 and 10:00 AM under standardized conditions after an overnight fast. Patients were instructed to avoid intensive exercise for 48h prior to the test. The post-intervention biopsies were performed 48h following the last training session. The skin at the puncture site was cleaned with 2% chlorhexidine in 70% isopropyl alcohol, and locally anaesthetized with 5 mL of 2% lignocaine, injected into the skin and subcutaneous tissue. Muscle samples were obtained from the vastus lateralis muscle (VL) using a percutaneous needle with suction applied (modified Bergstrom technique) [44]. VL biopsies were taken from the same leg but in different incision areas. Subcutaneous adipose tissue was obtained by surgical biopsy from the periumbilical area, 3–5 cm to the left or right of the navel [45]. Immediately after collection, tissues were visually inspected, and excess blood, connective tissue and fat (for muscle samples) were removed. Tissue samples were then processed according to appropriate protocols for downstream assays as described below.

### 2.4. Biochemical Measurements of Subjects

#### 2.4.1. Anthropometrics and Body Composition

Anthropometric measurements and body composition analyses were performed in the fasted state using a calibrated stadiometer SECA 264 (SECA, Hamburg, Germany) and electronic scale SECA 769 (SECA, Hamburg, Germany). BMI was calculated as body mass (kg) divided by height (m) squared. Whole-body dual-energy X-ray absorptiometry (DXA) scans were performed for body composition analysis, using Lunar iDXA (GE Healthcare, Chicago, IL, USA). The total amount of lean body mass (LBM), fat mass (FM) and visceral adipose tissue mass (VAT mass) were measured and expressed in kilograms (kg).

#### 2.4.2. OGTT and Laboratory Measurements

The OGTT procedure was conducted in accordance with ADA recommendations [21]. For each patient, the test commenced between 7:30–8:00 AM. The test started with baseline blood collection (0 min), followed by oral consumption of a solution of 75 g of glucose in 300 mL of room temperature water. Next, blood draws were performed 30, 60 and 120 min after the glucose administration for glucose and insulin measurements. Blood glucose was measured in plasma using the colorimetric method with Cobas c111 (Roche Diagnostics, Basel, Switzerland) according to manufacturer protocols. Insulin concentration was measured using the commercial immunoradiometric assay (IRMA) kit according to manufacturer protocols (DIAsource ImmunoAssays SA, Louvain-la-Neuve, Belgium).

On the day of the biopsy, venous blood was collected before starting the biopsy procedure to measure serum triglycerides (TG), total cholesterol (TChol), high-density lipoprotein cholesterol (HDL) and low-density lipoprotein cholesterol (LDL) concentrations, using colorimetric Cobas c111 kits according to manufacturer protocols (Roche Diagnostics, Basel, Switzerland). Haemoglobin A1c (HbA1c) was measured by the high-performance liquid chromatography (HPLC) kit according to manufacturer protocols (Bio-Rad VARIANT, Bio-Rad Laboratories, Hercules, CA, USA).

#### 2.4.3. Glucose Homeostasis Derivates

To evaluate insulin resistance, the homeostatic model assessment for insulin resistance was calculated: HOMA-IR = fasting insulin concentration (μIU/mL) × fasting glucose concentration (mmol/L) / 22.5. To evaluate beta cell function, the homeostatic model assessment of beta cell function was calculated: HOMA-β = (20 × fasting insulin concentration (μIU/mL) / (fasting glucose concentration (mmol/L) − 3.5) [46].

### 2.5. Exercise Testing

All subjects underwent an exercise test on a treadmill (Quasar Med, h/p/cosmos, Germany) using the progressive Balke standard protocol to measure maximal oxygen consumption (VO_2max_) [47]. The test was performed independently twice (Figure 1) to determine physical fitness and target workload to be used for endurance exercises of the intervention. During the test, patients wore silicone masks connected to a metabolic assay cart (Quark, Cosmed, Rome, Italy) and breath-by-breath exhalation was continuously analyzed to measure minute ventilation and expired air for CO_2_ and O_2_ fractions. Subjects exercised until at least two of the following test termination criteria were reached: subjects’ volitional fatigue, respiratory exchange ratio reached 1.1 or higher (RER ≥1.1), subjects reached predicted maximal HR (HRmax) (using formula HRmax = 220 − age) or oxygen uptake did not increase in spite of increasing workload (plateau). From these data, the maximal oxygen consumption (VO_2max_) was measured.

### 2.6. Mitochondria Measurements

Citrate synthase (CS) and β-hydroxy-acyl-CoA dehydrogenase (HAD) activity were measured as previously described in skeletal muscle [48]. In adipose tissue, minor modifications were made to the skeletal muscle protocol, and approximately 35 mg of adipose tissue was homogenized (2 × 2 min) for the analysis. After the homogenization the sample was left for 5 min on ice, followed by 2 min of centrifugation at 14,000× *g*. The stratified supernatant immediately inferior to the lipid layer was removed and frozen for subsequent analysis.

Mitochondrial respiratory capacity was measured in permeabilized skeletal muscle and adipose tissue. The methods have been described previously in skeletal muscle [49], and adipose tissue [50]. Briefly, skeletal muscle fibers were gently separated with sharp needles on ice in the preservation buffer (BIOPS, see [49]). Chemical permeabilization was done in BIOPS with saponin (50 µg/mL) for 30 min. This was followed by two washes (10 min) in mitochondrial respiration medium on ice (MiR05, see [50] for content). Finally, the fibers were weighed and added to the Oxygraph-2k (Oroboros, Innsbruck, Austria). Skeletal muscle experiments were performed in duplicate at 37 °C and under hyperoxic conditions (O_2_ concentration: 450–200 μM) to avoid oxygen diffusion limitations. Adipose tissue was gently dissected on ice to remove capillaries and connective tissue in the BIOPS buffer. This was followed by one wash (10 min) in MiR05, before the adipose tissue was weighed and added to the oxygraph. Adipose tissue experiments were run in duplicate at 37 °C and under normoxic conditions (O_2_ concentration: 200–100 μM). The following protocol was used for skeletal muscle as well as adipose tissue, with one minor difference. For the adipose tissue measurement digitonin (2.5 mg/mL) was added to the respiratory chamber before starting the analysis. Complex I-linked LEAK respiration (LEAK) was assessed with the NADH-generating substrate malate (2 mM). Hereafter, adenosine diphosphate (ADP) was added to the chamber by a saturating concentration (5 mM) and followed by the addition of glutamate (10 mM) to determine complex I-linked OXPHOS capacity (CI*P*). The integrity of the outer mitochondrial membrane was measured with cytochrome c (10 μM) and to determine maximal complex I + II-linked OXPHOS capacity, succinate (48 mM) was added (CI + II*P*). Succinate was added in 10 steps (0.1–0.2–0.5–1.0–2.0–4.0–8.0–16.0–24.0–48.0 mM) to measure the sensitivity for complex II-linked substrates. Finally, the uncoupler Carbonyl cyanide p-trifluoromethoxyphenylhydrazone (FCCP) was added in a stepwise manner (0.25 μM per step) to measure the maximal capacity of the electron transfer system (ETSmax). The addition of cytochrome c did not increase respiration in adipose tissue or skeletal muscle indicating that the integrity of the membrane was intact (results presented in Supplementary Figure S1).

Moreover, to investigate mitochondrial substrate handling we calculated the sensitivity of mitochondrial respiration in skeletal muscle using succinate. A non-linear regression (one site saturation equation) was used based on the individual raw data from the recordings of respiratory fluxes. The Michaelis-Menten constant (Km), numerically equal to the succinate concentration at which the flux is half of the maximum, was calculated.

For analysis of mitochondrial respiration, the oxygen flux was calculated from the derivative of the oxygen concentration of the chamber. To address the dissociation of mitochondrial content and respiration, the oxygen flux was normalized per tissue weight (mass-specific respiration) and per CS activity (mitochondria-specific respiration), which is a marker of mitochondrial content.

### 2.7. Statistical Analyses

All analyses were performed in R version 3.6.0 [51]. We tested all continuous response parameters for normality with Shapiro–Wilk tests as well as visual inspections of residuals where appropriate. If response arrays did not pass the Shapiro–Wilk test, the data were transformed by log_10_, square-root, exponential, or fraction transformations to achieve normality. Discrete parameters (i.e., diagnosis) are presented as frequencies (percentages) and continuous parameters as estimated marginals means (95% confidence interval). In order to calculate these least-squares means, we fit linear mixed models to normalized response data with time, diagnosis, and their interaction, as well as age, as fixed effects and patient identification number as a random effect. These models paired subjects as random effects, accounting for this experimental design in which the same subjects were resampled over time. To determine whether fixed effect terms were significant, we calculated *p*-values for each term by constructing Type III ANOVA tables with Satterthwaite’s Method. We conducted post-hoc tests on significant terms using pairwise comparisons among factor levels within significant terms. Significant post-hoc tests are denoted in figures and tables where appropriate. These significant values were corrected for multiple testing using the Benjamini–Hochberg (FDR) method in a family-wise manner, accounting for false discovery within analyses. To examine the correlational architecture of the data, we calculated matrices of pairwise Pearson’s correlations among parameter values at different time points, as well as among absolute changes from baseline to follow-up.

## 3. Results

### 3.1. Clinical Characteristic of Studied Groups and the Effectiveness of Exercise Intervention

Of 54 enrolled patients, 19 (35%) were normoglycemic; 27 (50%) were pre-diabetic with impaired fasting glucose, and 8 (15%) were pre-diabetic with both impaired fasting glucose and impaired glucose tolerance. The median age (IQR) was 47 years (43.0–50.8 years) and the median BMI at baseline was 28.81 kg/m^2^ (27–31.6 kg/m^2^). Age did not significantly differ among diagnosis groups with normoglycemics having an average age of 45.8 years (43.1–48.4 years), subjects with IFG 48.4 years (46.2–50.6 years), and subjects with IFG + IGT 50.1 years (46.1–54.2 years). Although these differences were not significant, they did show a suggestive trend of increasing dysglycemia with age, so we accounted for this trend by controlling for age as a covariate in subsequent models. The average daily calorie intake at baseline was 2092 kcal (1692–2554 kcal). Based on curated food diaries, mean daily protein intake was 88.9 g (74.9–113.7 g); mixed fats 69.6 g (59.1–92.6 g); and simple/complex carbohydrates 286.5 g (205.5–337.9 g). No significant differences in dietary parameters were found among the studied groups.

At baseline prior to the exercise intervention, we found that diagnosis correlated to differences in several clinical parameters. Specifically, there were significant differences in BMI, HbA1c, fasting and 2-h glucose and insulin concentrations, HOMA-IR and visceral fat mass. In all, normoglycemics showed the lowest values of these metrics and patients with combined IFG + IGT showed the highest (Table 2). The exercise intervention significantly improved the majority of measured clinical parameters including weight, BMI, glucose homeostasis parameters, fat and lean mass (Table 2), however, the improvements in lipids profile did not reach the level of significance.

Interestingly, most baseline differences among studied groups were driven by differences between NG vs. IFG + IGT, and the intervention eliminated most of the differences (i.e., making the NG group statistically indistinguishable from the IFG + IGT group). Indeed, following the intervention, only HbA1c, HOMA-IR and 2-h insulin concentration remained significantly different between NG vs. IFG + IGT. For IFG vs. IFG + IGT, we observed that baseline differences in glucose and insulin concentrations during OGTT and HOMA-IR were not significant anymore after the intervention, although 2-h insulin concentration remained significantly different (Supplementary Table S1). Moreover, we tested directly for differences in the response to exercise between studied groups. We found that there was significantly more improvement in fasting and 2-h glucose and insulin concentrations, as well as VO_2max_ and HOMA-IR in the IFG + IGT group, compared to the remaining groups (Table 2).

### 3.2. Mitochondria-Skeletal Muscle

**Figure 2:**
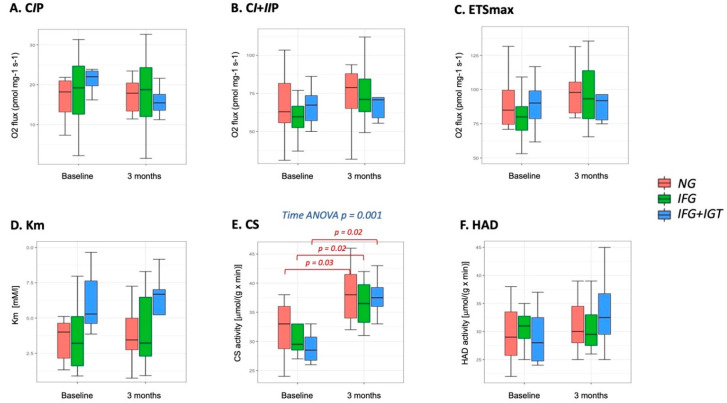
MUSCLE mitochondrial content (via citrate synthase activity) and mass-specific respiratory capacity parameters in patients with Normoglycemia (NG), isolated Impaired Fasting Glucose (IFG) and Impaired Fasting Glucose and Impaired Glucose Tolerance (IFG + IGT), before and after 3 months of exercise intervention. (**A**). Complex I-linked OXPHOS capacity (CIP); (**B**). Maximal complex I + II-linked OXPHOS capacity (CI + IIP), (**C**). maximal capacity of the electron transfer system (ETSmax); (**D**). Km for succinate titration, (**E**). Citrate Synthase (CS) activity. (**F**). β-hydroxy-acyl-CoA dehydrogenase (HAD) activity. Square root transformation was performed to calculate model-based means and 95% CI.

Prior to the initiation of the exercise intervention, we did not find any significant differences in mitochondrial respiratory capacity, sensitivity for succinate or citrate synthase nor HAD activity in skeletal muscle (both mass- and mitochondria-specific) among studied groups, indicating no difference in respiratory function or amount of mitochondria among diagnosis groups. Results of citrate synthase activity and mass-specific mitochondrial respiration are presented in Figure 2. Mitochondria-specific respiration parameters in skeletal muscle are presented in Supplementary Table S2.

Among parameters of mitochondrial respiration (again both mass- and mitochondria-specific) and content, we found that the exercise intervention caused significant improvement in citrate synthase activity (*p*-value = 0.001). However, we found no differences in the response to the exercise intervention among groups in all measured parameters in muscle tissue, indicating that all three studied groups responded similarly to the exercise intervention (Results with post-hoc *p*-values are presented in Figure 2).

**Figure 3:**
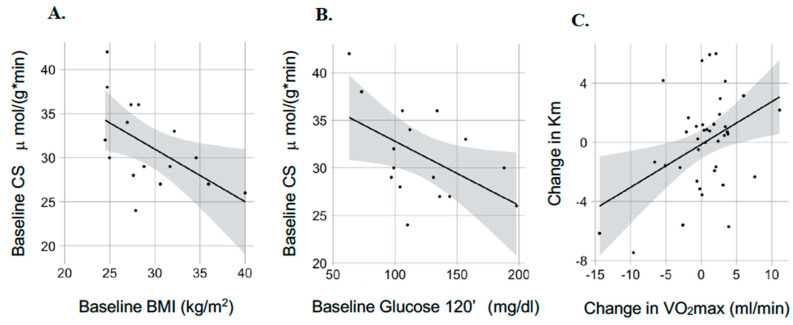
Pairwise correlations among clinical features and parameters of mitochondrial respiration in skeletal muscle. (**A**). Correlation between Citrate Synthase (CS) activity and Body Mass Index (BMI). (**B**). Correlation between Citrate Synthase (CS) activity and glucose concentration at 120-min of OGTT (glucose-120′). (**C**). Correlation between changes in Km for succinate titration and changes in VO_2max_ under the intervention.* means multiplication.

We also calculated pairwise correlations among mitochondrial and clinical parameters. We found that for baseline measures, there are significant correlations between CS activity and BMI (r2 = 0.55; *p* = 0.028) and CS activity and 2-h glucose (r2 = −0.51; *p* = 0.042) (See Figure 3A,B). Moreover, when we analyzed how changes in mitochondrial parameters under the exercise intervention correlate with improvements in clinical parameters, we discovered that changes in Km were positively correlated with VO_2max_ changes under the intervention (r2 = 0.4; *p* = 0.011) (see Figure 3C). These correlations are shown in Figure 3 with lines of best fit with 95% confidence intervals.

### 3.3. Mitochondria-Adipose Tissue

**Figure 4:**
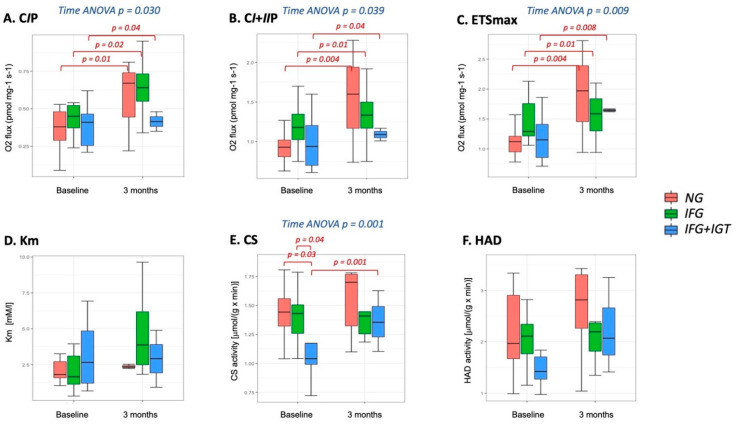
ADIPOSE TISSUE mitochondrial content (via citrate synthase activity) and mass-specific respiratory capacity parameters in patients with Normoglycemia (NG), isolated Impaired Fasting Glucose (IFG) and Impaired Fasting Glucose and Impaired Glucose Tolerance (IFG + IGT), before and after 3 months of exercise intervention. (**A**). Complex I-linked OXPHOS capacity (CIP); (**B**). Maximal complex I + II -linked OXPHOS capacity (CI + IIP), (**C**). maximal capacity of the electron transfer system (ETSmax); (**D**). Km for succinate titration, (**E**). Citrate Synthase (CS) activity. (**F**). β-hydroxy-acyl-CoA dehydrogenase (HAD) activity. Square root transformation was performed to calculate model-based means and 95% CI.

At baseline, we found significant differences in the citrate synthase activity: subjects with IFG + IGT had the lowest activity (*p* = 0.041), representing the lowest content of mitochondria in adipose tissue (Results with post-hoc *p*-values are presented in Figure 4).

In the response to exercise intervention, we observed significant improvement in mass-specific Complex I-linked LEAK respiration (*p* = 0.030), complex I + II -linked OXPHOS capacity (*p* = 0.039), the maximal capacity of the electron transfer system (*p* = 0.009)–see results, including post-hoc *p*-values in Figure 4. Similar results were observed for mitochondria-specific respiration parameters (presented in Supplementary Table S3). Moreover, there was a significant increase in the citrate synthase activity (*p*-value = 0.001), which eliminated differences between groups observed before the exercise intervention. We found no differences in the response to the exercise intervention among groups, indicating that all three studied groups responded in a similar way (Figure 4), which reflects the behavior of muscle tissue.

We also calculated how mitochondrial parameters correlate with clinical parameters. We found that for baseline measures, there are significant correlations for CS activity and the following clinical parameters: BMI (r2 = −0.45; *p* = 0.006), fasting (r2 = −0.40; *p* = 0.018) and 2-h glucose (r2 = −0.63; *p* < 0.001), HOMA-IR (r2 = −0.57; *p* = 0.001) and fat mass (r2 = −0.34; *p* = 0.048). Significant correlations were also observed for HAD activity and following parameters: BMI (r2 = −0.45; *p* = 0.007), 2-h glucose (r2 = −0.35; *p* = 0.037), VO_2max_ (r2 = 0.40; *p* = 0.017), HOMA-IR (r2 = −0.56; *p* = 0.001) and fat mass (r2 = −0.44; *p* = 0.008).

## 4. Discussion

In our study, we report that in subjects with prediabetes, skeletal muscle and adipose tissue mitochondrial respiration, both mass- and mitochondria-specific, are not altered, despite significantly lower content of these organelles in adipose tissue (assessed via citrate synthase activity) compared to age-, BMI- and VO2max-matched normoglycemic subjects. Moreover, we show that clinical improvements in metabolic parameters in response to exercise are accompanied by improvement in the amount of mitochondria, as assayed by increased citrate synthase activity, in both tissues.

We showed that the 3-month exercise intervention is effective in alleviating differences in glucose homeostasis and anthropometric parameters in both IFG and IFG + IGT relative to normoglycemia. This points to behavioral interventions as a primary treatment and prophylactic modality in the early stages of glucose metabolism dysregulation, which may prevent the development of overt type 2 diabetes. As expected, we observed significant differences between diagnosis groups in several clinical parameters related to patients’ dysglycemic status, where the more advanced glucose metabolism dysregulation was associated with the most pronounced difference compared to the normoglycemic group. Importantly, the exercise intervention was effective in all studied groups, but there were significant differences in how different groups responded to the intervention. However, these differences were not driven by “resistance” or diminished responsiveness in patients with prediabetes, but rather the opposite: subjects with both impaired fasting glucose and impaired glucose tolerance, with the highest risk of diabetes development, improved the most in glucose and anthropometric parameters. This is likely because these patients have the greatest “room for improvement,” and in fact, many nearly achieved similar levels of glycemic parameters as normoglycemic patients after just 3 months of increased physical activity.

The finding that some individuals have isolated IGT or IFG suggests that there are different pathophysiological mechanisms causing distinct defects in glucose homeostasis [52]. Prediabetes pathophysiology represents biological abnormalities occurring in the body before type 2 diabetes is fully developed. The pathophysiology of prediabetes is similar to overt type 2 diabetes since two main abnormalities are present in both: insulin resistance and diminished insulin secretion by pancreatic beta cells. However, each prediabetes “subtype,” IFG and IGT, has subtly different pathomechanistic etiologies, underscoring prediabetes’s status as a multifaceted disease entity with at least two separate pathologies of different origin and mechanisms leading to T2D development. They are simply united under the umbrella of their common insulin resistance phenotype. It has been hypothesized that individuals with isolated IFG have hepatic insulin resistance with abnormal hepatic glucose production [53,54,55], whereas those with isolated IGT predominantly have muscle insulin resistance and normal or slightly reduced hepatic insulin sensitivity. Moreover, as skeletal muscles contribute more to whole-body insulin sensitivity, compared to the liver, subjects with isolated IGT have on average 15–30% higher whole-body insulin resistance compared to those with isolated IFG [54,55,56]. Consequently, subjects with both IFG and IGT have both hepatic and muscle insulin resistance, which confers an increased risk of progressing to diabetes compared with having only one abnormality [20]. This concords with our results which show that in skeletal muscle tissue, there were no differences between NGT and IFG, potentially suggesting the primary role of liver insulin resistance in IFG patients.

A major aim of our study was to address how mitochondrial respiration and content may dissociate both during diabetes development and response to exercise in a prediabetes population. Studies have shown, that although related, these aspects of mitochondrial adaptation may be independent, especially in exercise adaptations [35]. Previous studies suggest that training volume is a crucial factor in the exercise-induced increases in mitochondrial content (measured both by microscopy and using proxy parameters like CS activity) and exercise intensity as the main driver of improvements in mitochondrial respiration [35].

Few studies have investigated mitochondrial adaptations in the prediabetic state. One study examined mitochondrial respiratory capacity in non-diabetic patients with increasing insulin resistance, finding differences between the groups in mitochondrial respiratory capacity [57] but none in mitochondrial content (measured as mtDNA content). When focusing on mitochondrial function in patients with type 2 diabetes, some studies indicate mitochondrial dysfunction in patients with type 2 diabetes [4,10], but a consensus remains lacking in the literature [13,58,59]. In order to be able to investigate differences in mitochondrial respiratory capacity and content between patients with type 2 diabetes and matched controls, it seems crucially important to recruit subjects with similar activity levels, since it is known that physical activity influences mitochondrial function [28]. We did not observe baseline differences in either mitochondrial content (using CS activity as a proxy) or respiration (both mass- and mitochondria-specific) in skeletal muscle of patients with prediabetes, compared to normoglycemic subjects. This was a surprising finding, but upon analysis of the clinical characteristic of our subjects, we observed that they were well-matched in parameters influencing mitochondrial function, especially VO2max. Together, this suggests that inactivity and low exercise capacity, rather than dysglycemia, are the main drivers of mitochondrial differences in prediabetic subjects. Previous studies have shown that patients with dysglycemia (insulin resistance, prediabetes, T2D) have impaired mitochondrial respiratory capacity, and/or mitochondrial content in skeletal muscles, compared to healthy controls [4,6,7,8,9,10]. In general, these control groups differ in many potentially causal factors from the prediabetic groups, confounding specific inferences based on glycemic status alone. By contrast, in our study, we compare our prediabetic subjects to a normoglycemic group that significantly differs only in glycemic parameters, but no obesity or VO2maix. Our findings suggest that previous studies may have detected differences in mitochondrial respiration and amount due to pre-existing differences in physical fitness, rather than dysglycemia per se, which is in line with a previous study by Larsen et al. [13].

Interestingly, although we did not expect marked differences in mitochondrial parameters in adipose tissue between studied groups, we found that the CS activity was reduced in IFG + IGT in the adipose tissue at baseline. A previous study investigated mitochondrial respiratory capacity and content in adipose tissue from obese patients with and without type 2 diabetes and found no changes in respiratory capacity and content (quantified by mtDNA copy numbers) [60]. The exercise intervention resulted in an increase in citrate synthase activity. These results are not in line with studies published previously, in which no changes were observed in mitochondrial content (mtDNA or citrate synthase activity) [48,61]. These studies were of shorter duration and under less strenuous exercise interventions, which could explain this discrepancy. In a study by Larsen and colleagues, no changes were seen in mitochondrial respiratory capacity, which is in line with results from the present study (for both mass- and mitochondria-specific respiration). If citrate synthase is a good marker of mitochondrial content in adipose tissue, it could be speculated that mitochondrial content increases prior to mitochondrial respiratory capacity. A potential molecular mechanism underlying the findings in this study could rest on the so-called “browning” of white adipose tissue, which is the switch from white adipocytes to brown-like adipocytes (beige adipocytes) produced by an increase in mitochondrial biogenesis. Previous studies, notably in mouse models, have shown that exercise induces WAT browning and glucose homeostasis improvements [62]

Across the literature, there are a number of conflicting results as to whether T2D and insulin resistance developments are causes or results of dysfunction of mitochondria (OXPHOS) or the reduced amount of these organelles in metabolically active tissue [8,9,34]. In our study, we address these alternatives by showing that in the early stages of T2D development, the content of mitochondria may play a more important role than function. However, we must emphasize that these differences are present only when impaired fasting glucose is accompanied by impaired glucose tolerance, the more advanced step of dysglycemia. Indeed, this occurs despite pre-existing significant clinical differences among NGT, IFG and IGT groups, indicating that early stages of diabetes development may not involve mitochondria in muscle and suggests an important role for other tissues like the liver.

Another important finding of our study is that mitochondria are important mediators of exercise effectiveness in prediabetic subjects. We show that differences in the amount of mitochondria (expressed as CS activity) between studied groups are eliminated after the exercise intervention. This significant change occurred in the direction of more mitochondria in both muscle and adipose tissue. Moreover, even if not significant, we see clear trends in nearly all studied parameters of oxidative phosphorylation that exercise improves mitochondrial function. The available literature on mitochondrial function in patients with prediabetes, and its response to an exercise intervention remains limited. Most studies focus on subjects with extant type 2 diabetes, rather than a prediabetes cohort. These studies, on type 2 diabetes or obese patients, have shown improvements in mitochondrial respiratory capacity and content after exercise interventions. By contrast, we demonstrate an increase in mitochondrial content, via increased activity of CS in both adipose tissue and skeletal muscle, but a lack of improvement in mitochondrial respiratory capacity (both mass- and mitochondria-specific) measurements in skeletal muscle. Consistent with our study, Hoffmann et al. show the effect of an endurance exercise intervention on mitochondrial parameters in obese subjects, which is overall a similar cohort as in our study. Even though they show improvements in mitochondrial respiration (not normalized to mitochondrial content), they correctly point out that a parallelly observed increase in mitochondrial content is likely a reason for the increase in mitochondrial respiration.

The observed dissociation of mitochondrial content and respiration in our study, where we show improvements in CS activity but no changes in either mass- or mitochondria-specific respiration, is consistent with other studies and should not be interpreted as surprising. We speculate that this is related to the specific volume and intensity of our intervention. As summarized by Granata et al. [35], training volume may be a key determinant of changes in mitochondrial content, and exercise intensity may be the main determinant of changes in mitochondrial respiration in response to the exercise intervention. Studies have shown that by applying different volumes and intensities of exercise, different combinations of mitochondrial content and respiration may result. For example, Meinild Lundby et al. [63] have shown an increase in mitochondrial content but no change in mass- and mitochondria-specific respiration. Similar results but combined with a reduction in mitochondrial respiration adjusted to mitochondrial content were observed by Montero et al. [38]. In both studies, a moderate-intensity exercise intervention was applied. These results are in line with our finding; where, in response to the 12-week intervention with three sessions per week and the intensity of 60–70% of individual’s VO2max for endurance and 60–75% of 1 Repetition Max for resistance exercises, caused a dissociated response of improved citrate activity but not in mass- and mitochondria-specific parameters. Montero et al. hypothesize that this might be caused by the transitory normalization of OXPHOS in skeletal muscle in the presence of increased oxygen delivery [38]. However, the mechanism responsible for these adaptations is beyond the scope of this study and requires further in-depth investigation.

Moreover, it is important to note a unique feature of our study: the specifics of the mode of the intervention. A vast majority of the studies on mitochondrial respiratory parameters and content focuses on one mode of exercise: endurance or strength. In our study, we use a combination of both modes, which was shown to be the most effective in diabetes prevention and treatment [42,43,63]. Studies showing improvements in mitochondrial respiratory capacity are mostly studied in response to endurance exercise interventions, and the understanding of response to strength or combined exercise modes remains sharply limited, mostly focusing on T2D patients [64,65]. In our study, we explore mitochondrial respiratory capacity and content in both skeletal muscle and adipose tissue, on precisely specified prediabetes status, simultaneously measuring mitochondrial respiratory capacity and content, in response to combined endurance and strength exercise intervention, which makes our study novel.

It is especially pressing that patients with prediabetes are treated so as to prevent the development of diabetes. Studies have shown a key role of mitochondrial function and amount in both skeletal muscle and adipose tissue in preventing the progression of T2D. Due to the duration of this study which followed patients for 3 months, it was not possible to draw direct conclusions as to the prevention of T2D development since the onset of diabetes may occur over many years. However, it was previously shown that two-hour glucose concentration during OGTT is a strong predictive feature of T2D progression in subjects with prediabetes [66,67]. Furthermore, we show that it was correlated with increased activity of citrate synthase, thus implicating mitochondrial content as an important mediator of T2D prevention. Supporting this view, other studies have shown that the mitochondrial density in skeletal muscle is decreased in obese subjects with insulin resistance [1,5] and may be an important aspect of diabetes development. This is consistent with our findings where we show that mitochondrial content in subjects with prediabetes (especially IFG+IGT) is reduced in adipose tissue and improves upon exercise which helped to restore these differences, presumably reducing the risk of progression to T2D.

From the clinical perspective, it is crucial to determine which mitochondrial adaptation, content, respiration, or a combination of both, provides optimal metabolic outcomes. This should be further investigated in studies comparing responses to moderate- and high-intensity training with different training volumes. A better understanding of these mechanisms will help to identify the optimal exercise prescription to prevent and treat diabetes and other metabolic disorders.

This study has several limitations. The sample size is not large, which might result in our inability to detect smaller effects. However, this was a rigorous, highly supervised exercise intervention and it is logistically difficult to scale up such an intense effort to a large number of participants. Another important limitation is that the “mitochondrial parameters” presented here address only one aspect of the function of these organelles, which is their role in oxidative phosphorylation. Of course, mitochondria participate in many other metabolically important activities, such as ROS production, the maintenance of transmembrane electric potential, fusion and fission of mitochondria, and apoptosis-programmed cell death. These processes were beyond the scope of this study, but likely play important roles in the progression of dysglycemia and thus be cryptic mechanisms of exercise effects. Moreover, as described in the introduction, the CS was used as a biomarker of mitochondrial content, since it is a proxy metric of the transmission electron microscopy-based measurement of mitochondrial volume density (MitoVD), which is the gold-standard technique for the assessment of mitochondrial content. Furthermore, we selected anatomic locations for the biopsy, that is most commonly sampled in other studies so that our results are more directly comparable to these studies. However, it should be emphasized that there is bulk-tissue and molecular heterogeneity in both skeletal muscles and adipose tissue depots [68,69], so drawing conclusions based on our and others’ findings with regards to the whole body must be taken with caution. Additionally, in this study, only male subjects participated in the exercise intervention, and our conclusions are limited to this sex. Further studies are needed to explore whether differences between sexes in mitochondrial parameters exist in patients with dysglycemia. Finally, our study does not include a control group (without exercise intervention), and thus, there is a small possibility that confounding variables correlated to the detected exercise effects.

## 5. Conclusions

In conclusion, we show that in the early stages of type 2 diabetes development, there are no differences in the mitochondrial respiration and content (via CS activity) in skeletal muscle, but there is a significantly reduced number of mitochondria in the adipose tissue of patients with dysglycemia. We speculate that this may point to a predominant role for hepatic insulin resistance in the early stages of T2D development, but this will require further study. Moreover, we also show that clinical metabolic improvements under the exercise parallel increased amounts of mitochondria both in skeletal muscle and adipose tissue, rather than improvements in oxidative phosphorylation.

## Figures and Tables

**Table 1:** Inclusion and exclusion criteria for study participants.

Inclusion Criteria	Exclusion Criteria
Age: 35–65 years old BMI: 25–35 kg/m^2^ Male gender Ability of performing exercise training Sedentary lifestyle assessed using the Polish version of International Physical Activity Questionnaire—Long Form (IPAQ-LF) [40]	Smoking Drug or alcohol addiction Any chronic disease (exceptions: hypertension, obesity with BMI ≤ 35 kg/m^2^, type 2 diabetes) Any chronic medications (exceptions: angiotensin-converting-enzyme inhibitors for hypertension and metformin in type 2 diabetics) Highly active lifestyle Medical contraindications to participate in planned exercise sessions

**Table 2:** Clinical characteristics of studied participants. Presented are mean values and interquartile ranges for each parameter. IFG-subjects with impaired fasting glucose and normal glucose tolerance; IFG+IGT–subjects with impaired fasting glucose and impaired glucose tolerance; BMI–body mass index; HbA1c-Haemoglobin A1c; VAT–visceral adipose tissue; TG–triglycerides; HDL-high-density lipoprotein cholesterol; LDL-low-density lipoprotein cholesterol; VO2max-maximal oxygen consumption; HOMA-IR-homeostatic model assessment for insulin resistance was calculated; HOMA-β-homeostatic model assessment of beta-cell function. Bold and Italic values denote statistical significance at the *p* < 0.05 level.

Parameter (Unit)	Normoglycemia *n* = 19		IFG *n* = 27		IFG + IGT *n* = 8		*p*-Value		
	Before	After 3 m.	Before	After 3 m.	Before	After 3 m.	Baseline	Time	Time × Diagnosis
**Weight** **(kg)**	89.43	89.30	92.80	91.69	95.60	93.57	0.557	*0.020*	0.272
	(83.84–95.02)	(83.70–94.89)	(88.20–97.40)	(87.09–96.30)	(87.07–104.14)	(85.01–102.13)			
**BMI** **(kg/m^2^)**	27.62	27.55	29.37	29.00	31.98	31.31	*0.021*	*0.012*	0.288
	(26.08–29.17)	(26.00–29.09)	(28.09–30.64)	(27.73–30.28)	(29.62–34.34)	(28.94–33.69)			
**HbA1c** **(%)**	5.24	5.12	5.46	5.31	5.76	5.61	*0.008*	*<0.001*	0.881
	(5.06–5.42)	(4.94–5.30)	(5.32–5.61)	(5.16–5.46)	(5.49–6.03)	(5.33–5.88)			
**Glucose 0′** **(mg/dL)**	96.14	101.19	109.50	105.26	120.19	110.70	*<0.001*	0.148	*<0.001*
	(99.15–93.32)	(104.60–98)	(112.72–106.46)	(108.29–102.40)	(127.59–113.60)	(117.36–104.76)			
**Glucose 120′ (mg/dL)**	85.76	98.79	96.10	92.09	165.88	116.15	*<0.001*	*0.006*	*<0.001*
	(75.14–97.08)	(87.19–111.11)	(86.74–105.93)	(82.82–101.86)	(143.36–190.04)	(96.61–137.47)			
**Insulin 0′** **(μU/mL)**	9.44	9.28	12.32	10.37	21.07	15.60	*<0.001*	*<0.001*	*0.015*
	(7.23–11.66)	(7.06–11.49)	(10.48–14.15)	(8.54–12.21)	(17.28–24.87)	(11.62–19.57)			
**Insulin 120′** **(μU/mL)**	18.23	19.79	36.36	22.69	178.29	82.96	*<0.001*	*0.003*	*0.014*
	(12.13–27.40)	(13.09–29.91)	(25.99–50.87)	(16.16–31.87)	(88.88–357.62)	(39.9–172.50)			
**VAT mass** **(kg)**	1.55	1.46	1.80	1.57	2.59	2.19	*0.030*	*<0.001*	0.075
	(1.24–1.88)	(1.16–1.79)	(1.52–2.09)	(1.32–1.85)	(2.00–3.26)	(1.65–2.82)			
**Total Chol. (mg/dL)**	193.29	194.11	194.64	200.66	206.29	196.01	0.879	0.762	0.258
	(175.80–210.79)	(176.48–211.74)	(180.22–209.06)	(186.15–215.18)	(179.56–233.02)	(168.67–223.35)			
**TG** **(mg/dL)**	98.44	89.79	102.07	104.72	151.66	125.88	0.166	0.086	0.184
	(78.50–123.45)	(71.48–112.79)	(84.70–123.01)	(86.80–126.35)	(107.32–214.33)	(88.39–179.28)			
**HDL** **(mg/dL)**	56.20	57.19	50.54	50.27	53.33	52.38	0.258	0.933	0.745
	(50.25–62.49)	(51.16–63.57)	(45.87–55.43)	(45.60–55.17)	(44.62–62.82)	(43.62–61.94)			
**LDL** **(mg/dL)**	122.09	124.88	131.03	131.84	130.72	134.24	0.684	0.509	0.936
	(106.35–137.82)	(109.01–140.75)	(118.06–144.00)	(118.77–144.90)	(106.67–154.77)	(109.59–158.89)			
**VO_2max_ (mL/kg/min)**	32.33	32.65	30.44	32.20	29.07	31.26	0.427	0.221	*0.009*
	(31.18–35.48)	(31.46–35.83)	(28.66–32.22)	(30.40–34.00)	(25.78–32.36)	(27.85–34.68)			
**HOMA-IR**	2.07	2.13	3.13	2.46	5.95	4.25	*<0.001*	*0.006*	*0.030*
	(1.70–2.53)	(1.75–2.60)	(2.66–3.69)	(2.09–2.90)	(4.23–8.37)	(2.96–6.09)			
**HOMA-b** **(%)**	95.75	80.84	90.42	81.05	130.24	105.04	0.146	*0.007*	0.729
	(79.92–114.71)	(67.47–96.85)	(77.88–105.00)	(69.80–94.11)	(95.58–177.46)	(75.69–145.77)			
**Fat mass** **(kg)**	25.17	24.49	28.01	26.02	31.33	28.62	0.237	*<0.001*	0.136
	(21.84–28.51)	(21.14–27.83)	(25.26–30.76)	(23.26–28.77)	(26.24–36.43)	(23.49–33.75)			
**Lean mass** **(kg)**	60.46	61.64	61.18	62.39	59.78	60.74	0.835	*<0.001*	0.883
	(57.30–63.61)	(58.48–64.80)	(58.58–63.78)	(59.79–65.00)	(54.96–64.61)	(55.91–65.57)			

## Data Availability

The data that support the findings of this study are available from the corresponding author, (L.S.), upon request.

## Supplementary Materials

The following are available online at https://www.mdpi.com/article/10.3390/cells10113013/s1, Table S1: Differences between studied groups before the initiation and after completion of the exercise intervention. Table S2. MUSCLE mass-specific and mitochondria-specific respiratory capacity parameters in patients with Normoglycemia (NG), isolated Impaired Fasting Glucose (IFG) and Impaired Fasting Glucose and Impaired Glucose Tolerance (IFG + IGT), before and after 3 months of exercise intervention. Table S3. ADIPOSE TISSUE mass-specific and mitochondria-specific respiratory capacity parameters in patients with Normoglycemia (NG), isolated Impaired Fasting Glucose (IFG) and Impaired Fasting Glucose and Impaired Glucose Tolerance (IFG + IGT), before and after 3 months of exercise intervention. Figure S1: Changes in mitochondrial respiration after addition of Cytochrome C in adipose tissue and skeletal muscle.

## References

[1] Patti M.E., Corvera S. (2010). The Role of Mitochondria in the Pathogenesis of Type 2 Diabetes. Endocr. Rev..

[2] Petersen K.F., Befroy D., Dufour S., Dziura J., Ariyan C., Rothman D.L., DiPietro L., Cline G.W., Shulman G.I. (2003). Mitochondrial Dysfunction in the Elderly: Possible Role in Insulin Resistance. Science.

[3] Petersen K.F., Dufour S., Befroy D., Garcia R., Shulman G.I. (2004). Impaired Mitochondrial Activity in the Insulin-Resistant Offspring of Patients with Type 2 Diabetes. N. Engl. J. Med..

[4] Mogensen M., Sahlin K., Fernstrom M., Glintborg D., Vind B.F., Beck-Nielsen H., Hojlund K. (2007). Mitochondrial Respiration Is Decreased in Skeletal Muscle of Patients with Type 2 Diabetes. Diabetes.

[5] Ritov V.B., Menshikova E.V., He J., Ferrell R.E., Goodpaster B.H., Kelley D.E. (2005). Deficiency of Subsarcolemmal Mitochondria in Obesity and Type 2 Diabetes. Diabetes.

[6] Chomentowski P., Coen P.M., Radikova Z., Goodpaster B.H., Toledo F.G. (2011). Skeletal Muscle Mitochondria in Insulin Resistance: Differences in Intermyofibrillar versus Subsarcolemmal Subpopulations and Relationship to Metabolic Flexibility. J. Clin. Endocrinol. Metab..

[7] Schrauwen-Hinderling V.B., Kooi M.E., Hesselink M.K., Jeneson J.A., Backes W.H., van Echteld C.J., van Engelshoven J.M., Mensink M., Schrauwen P. (2007). Impaired in Vivo Mitochondrial Function but Similar Intramyocellular Lipid Content in Patients with Type 2 Diabetes Mellitus and BMI-Matched Control Subjects. Diabetologia.

[8] Kelley D.E., He J., Menshikova E.V., Ritov V.B. (2002). Dysfunction of Mitochondria in Human Skeletal Muscle in Type 2 Diabetes. Diabetes.

[9] Boushel R., Gnaiger E., Schjerling P., Skovbro M., Kraunsoe R., Dela F. (2007). Patients with Type 2 Diabetes Have Normal Mitochondrial Function in Skeletal Muscle. Diabetologia.

[10] Phielix E., Schrauwen-Hinderling V.B., Mensink M., Lenaers E., Meex R., Hoeks J., Kooi M.E., Moonen-Kornips E., Sels J.P., Hesselink M.K. (2008). Lower Intrinsic ADP-Stimulated Mitochondrial Respiration Underlies in Vivo Mitochondrial Dysfunction in Muscle of Male Type 2 Diabetic Patients. Diabetes.

[11] Stump C.S., Short K.R., Bigelow M.L., Schimke J.M., Nair K.S. (2003). Effect of Insulin on Human Skeletal Muscle Mitochondrial ATP Production, Protein Synthesis, and MRNA Transcripts. Proc. Natl. Acad. Sci. USA.

[12] Szendroedi J., Schmid A.I., Chmelik M., Toth C., Brehm A., Krssak M., Nowotny P., Wolzt M., Waldhausl W., Roden M. (2007). Muscle Mitochondrial ATP Synthesis and Glucose Transport/Phosphorylation in Type 2 Diabetes. PLoS Med..

[13] Larsen S., Stride N., Hey-Mogensen M., Hansen C.N., Andersen J.L., Madsbad S., Worm D., Helge J.W., Dela F. (2011). Increased Mitochondrial Substrate Sensitivity in Skeletal Muscle of Patients with Type 2 Diabetes. Diabetologia.

[14] Koves T.R., Ussher J.R., Noland R.C., Slentz D., Mosedale M., Ilkayeva O., Bain J., Stevens R., Dyck J.R.B., Newgard C.B. (2008). Mitochondrial Overload and Incomplete Fatty Acid Oxidation Contribute to Skeletal Muscle Insulin Resistance. Cell Metab..

[15] Goodpaster B.H., Sparks L.M. (2017). Review Metabolic Flexibility in Health and Disease. Cell Metab..

[16] Simoneau J.A., Kelley D.E. (1997). Altered Glycolytic and Oxidative Capacities of Skeletal Muscle Contribute to Insulin Resistance in NIDDM. J. Appl. Physiol..

[17] Ukropcova B., Sereda O., de Jonge L., Bogacka I., Nguyen T., Xie H., Bray G.A., Smith S.R. (2007). Family History of Diabetes Links Impaired Substrate Switching and Reduced Mitochondrial Content in Skeletal Muscle. Diabetes.

[18] Meex R.C., Schrauwen-Hinderling V.B., Moonen-Kornips E., Schaart G., Mensink M., Phielix E., van de Weijer T., Sels J.P., Schrauwen P., Hesselink M.K. (2010). Restoration of Muscle Mitochondrial Function and Metabolic Flexibility in Type 2 Diabetes by Exercise Training Is Paralleled by Increased Myocellular Fat Storage and Improved Insulin Sensitivity. Diabetes.

[19] Lee H., Song W. (2018). Exercise and Mitochondrial Remodeling in Skeletal Muscle in Type 2 Diabetes. J. Obes. Metab. Syndr..

[20] Tabák A.G., Herder C., Rathmann W., Brunner E.J., Kivimäki M. (2012). Prediabetes: A High-Risk State for Diabetes Development. Lancet.

[21] American Diabetes Association (2020). 2. Classification and Diagnosis of Diabetes: Standards of Medical Care in Diabetes-2020. Diabetes Care.

[22] Gillett M., Royle P., Snaith A., Scotland G., Poobalan A., Imamura M., Black C., Boroujerdi M., Jick S., Wyness L. (2012). Non-Pharmacological Interventions to Reduce the Risk of Diabetes in People with Impaired Glucose Regulation: A Systematic Review and Economic Evaluation. Health Technol. Assess..

[23] Balk E.M., Earley A., Raman G., Avendano E.A., Pittas A.G., Remington P.L. (2015). Combined Diet and Physical Activity Promotion Programs to Prevent Type 2 Diabetes Among Persons at Increased Risk: A Systematic Review for the Community Preventive Services Task Force. Ann. Intern. Med..

[24] Hemmingsen B., Gimenez-Perez G., Mauricio D., Roque I.F.M., Metzendorf M.I., Richter B. (2017). Diet, Physical Activity or Both for Prevention or Delay of Type 2 Diabetes Mellitus and Its Associated Complications in People at Increased Risk of Developing Type 2 Diabetes Mellitus. Cochrane Database Syst. Rev..

[25] Phielix E., Meex R., Moonen-Kornips E., Hesselink M.K., Schrauwen P. (2010). Exercise Training Increases Mitochondrial Content and Ex Vivo Mitochondrial Function Similarly in Patients with Type 2 Diabetes and in Control Individuals. Diabetologia.

[26] Trevellin E., Scorzeto M., Olivieri M., Granzotto M., Valerio A., Tedesco L., Fabris R., Serra R., Quarta M., Reggiani C. (2014). Exercise Training Induces Mitochondrial Biogenesis and Glucose Uptake in Subcutaneous Adipose Tissue through ENOS-Dependent Mechanisms. Diabetes.

[27] Boyle K.E., Friedman J.E., Janssen R.C., Underkofler C., Houmard J.A., Rasouli N. (2017). Metabolic Inflexibility with Obesity and the Effects of Fenofibrate on Skeletal Muscle Fatty Acid Oxidation. Horm. Metab. Res..

[28] Van Tienen F.H.J., Praet S.F.E., De Feyter H.M., Van Den Broek N.M., Lindsey P.J., Schoonderwoerd K.G.C., De Coo I.F.M., Nicolay K., Prompers J.J., Smeets H.J.M. (2012). Physical Activity Is the Key Determinant of Skeletal Muscle Mitochondrial Function in Type 2 Diabetes. J. Clin. Endocrinol. Metab..

[29] Fabbri E., Chia C.W., Spencer R.G., Fishbein K.W., Reiter D.A., Cameron D., Zane A.C., Moore Z.A., Gonzalez-Freire M., Zoli M. (2017). Insulin Resistance Is Associated with Reduced Mitochondrial Oxidative Capacity Measured by 31P-Magnetic Resonance Spectroscopy in Participants without Diabetes from the Baltimore Longitudinal Study of Aging. Diabetes.

[30] Montero D., Lundby C. (2017). Refuting the Myth of Non-Response to Exercise Training: ‘Non-Responders’ Do Respond to Higher Dose of Training. J. Physiol..

[31] Hoppeler H., Howald H., Conley K., Lindstedt S.L., Claassen H., Vock P., Weibel E.R. (1985). Endurance Training in Humans: Aerobic Capacity and Structure of Skeletal Muscle. J. Appl. Physiol..

[32] Daussin F.N., Zoll J., Dufour S.P., Ponsot E., Lonsdorfer-Wolf E., Doutreleau S., Mettauer B., Piquard F., Geny B., Richard R. (2008). Effect of Interval versus Continuous Training on Cardiorespiratory and Mitochondrial Functions: Relationship to Aerobic Performance Improvements in Sedentary Subjects. Am. J. Physiol. Regul. Integr. Comp. Physiol..

[33] Tonkonogi M., Walsh B., Svensson M., Sahlin K. (2000). Mitochondrial Function and Antioxidative Defence in Human Muscle: Effects of Endurance Training and Oxidative Stress. J. Physiol..

[34] Larsen S., Nielsen J., Hansen C.N., Nielsen L.B., Wibrand F., Stride N., Schroder H.D., Boushel R., Helge J.W., Dela F. (2012). Biomarkers of Mitochondrial Content in Skeletal Muscle of Healthy Young Human Subjects. J. Physiol..

[35] Granata C., Jamnick N.A., Bishop D.J. (2018). Training-Induced Changes in Mitochondrial Content and Respiratory Function in Human Skeletal Muscle. Sports Med..

[36] Granata C., Oliveira R.S.F., Little J.P., Renner K., Bishop D.J. (2016). Mitochondrial Adaptations to High-Volume Exercise Training Are Rapidly Reversed after a Reduction in Training Volume in Human Skeletal Muscle. FASEB J..

[37] Granata C., Oliveira R.S.F., Little J.P., Renner K., Bishop D.J. (2016). Training Intensity Modulates Changes in PGC-1α and P53 Protein Content and Mitochondrial Respiration, but Not Markers of Mitochondrial Content in Human Skeletal Muscle. FASEB J..

[38] Montero D., Cathomen A., Jacobs R.A., Flück D., de Leur J., Keiser S., Bonne T., Kirk N., Lundby A.K., Lundby C. (2015). Haematological Rather than Skeletal Muscle Adaptations Contribute to the Increase in Peak Oxygen Uptake Induced by Moderate Endurance Training. J. Physiol..

[39] Jacobs R.A., Lundby C. (2013). Mitochondria Express Enhanced Quality as Well as Quantity in Association with Aerobic Fitness across Recreationally Active Individuals up to Elite Athletes. J. Appl. Physiol..

[40] Craig C.L., Marshall A.L., Sjöström M., Bauman A.E., Booth M.L., Ainsworth B.E., Pratt M., Ekelund U., Yngve A., Sallis J.F. (2003). International Physical Activity Questionnaire: 12-Country Reliability and Validity. Med. Sci. Sports Exerc..

[41] Grontved A., Rimm E.B., Willett W.C., Andersen L.B., Hu F.B. (2012). A Prospective Study of Weight Training and Risk of Type 2 Diabetes Mellitus in Men. Arch. Intern. Med..

[42] Cuff D.J., Meneilly G.S., Martin A., Ignaszewski A., Tildesley H.D., Frohlich J.J. (2003). Effective Exercise Modality to Reduce Insulin Resistance in Women with Type 2 Diabetes. Diabetes Care.

[43] Sigal R.J., Kenny G.P., Boule N.G., Wells G.A., Prud’homme D., Fortier M., Reid R.D., Tulloch H., Coyle D., Phillips P. (2007). Effects of Aerobic Training, Resistance Training, or Both on Glycemic Control in Type 2 Diabetes: A Randomized Trial. Ann. Intern. Med..

[44] Shanely R.A., Zwetsloot K.A., Triplett N.T., Meaney M.P., Farris G.E., Nieman D.C. (2014). Human Skeletal Muscle Biopsy Procedures Using the Modified Bergstrom Technique. J. Vis. Exp..

[45] Chachopoulos V., Dinas P.C., Chasioti M., Jamurtas A.Z., Koutedakis Y., Flouris A.D. (2017). A Technique for Subcutaneous Abdominal Adipose Tissue Biopsy via a Nondiathermy Method. J. Vis. Exp..

[46] Matthews D.R., Hosker J.P., Rudenski A.S., Naylor B.A., Treacher D.F., Turner R.C. (1985). Homeostasis Model Assessment: Insulin Resistance and Beta-Cell Function from Fasting Plasma Glucose and Insulin Concentrations in Man. Diabetologia.

[47] Balke B., Ware R.W. (1959). An Experimental Study of Physical Fitness of Air Force Personnel. US Armed Forces Med. J..

[48] Larsen S., Danielsen J.H., Søndergård S.D., Søgaard D., Vigelsoe A., Dybboe R., Skaaby S., Dela F., Helge J.W. (2015). The Effect of High-Intensity Training on Mitochondrial Fat Oxidation in Skeletal Muscle and Subcutaneous Adipose Tissue. Scand. J. Med. Sci. Sports.

[49] Pesta D., Gnaiger E. (2012). High-Resolution Respirometry: OXPHOS Protocols for Human Cells and Permeabilized Fibers from Small Biopsies of Human Muscle. Methods Mol. Biol..

[50] Kraunsøe R., Boushel R., Hansen C.N., Schjerling P., Qvortrup K., Støckel M., Mikines K.J., Dela F. (2010). Mitochondrial Respiration in Subcutaneous and Visceral Adipose Tissue from Patients with Morbid Obesity. J. Physiol..

[51] R Core Team R Core Team (2014). R: A Language and Environment for Statistical Computing.

[52] Nathan D.M., Davidson M.B., DeFronzo R.A., Heine R.J., Henry R.R., Pratley R., Zinman B., American Diabetes A. (2007). Impaired Fasting Glucose and Impaired Glucose Tolerance: Implications for Care. Diabetes Care.

[53] Abdul-Ghani M.A., Jenkinson C.P., Richardson D.K., Tripathy D., DeFronzo R.A. (2006). Insulin Secretion and Action in Subjects with Impaired Fasting Glucose and Impaired Glucose Tolerance: Results from the Veterans Administration Genetic Epidemiology Study. Diabetes.

[54] Faerch K., Vaag A., Holst J.J., Glumer C., Pedersen O., Borch-Johnsen K. (2008). Impaired Fasting Glycaemia vs Impaired Glucose Tolerance: Similar Impairment of Pancreatic Alpha and Beta Cell Function but Differential Roles of Incretin Hormones and Insulin Action. Diabetologia.

[55] Meyer C., Pimenta W., Woerle H.J., Van Haeften T., Szoke E., Mitrakou A., Gerich J. (2006). Different Mechanisms for Impaired Fasting Glucose and Impaired Postprandial Glucose Tolerance in Humans. Diabetes Care.

[56] Festa A., D’Agostino R., Hanley A.J., Karter A.J., Saad M.F., Haffner S.M. (2004). Differences in Insulin Resistance in Nondiabetic Subjects with Isolated Impaired Glucose Tolerance or Isolated Impaired Fasting Glucose. Diabetes.

[57] Chanseaume E., Barquissau V., Salles J., Aucouturier J., Patrac V., Giraudet C., Gryson C., Duché P., Boirie Y., Chardigny J.M. (2010). Muscle Mitochondrial Oxidative Phosphorylation Activity, but Not Content, Is Altered with Abdominal Obesity in Sedentary Men: Synergism with Changes in Insulin Sensitivity. J. Clin. Endocrinol. Metab..

[58] Larsen S., Ara I., Rabøl R., Andersen J.L., Boushel R., Dela F., Helge J.W. (2009). Are Substrate Use during Exercise and Mitochondrial Respiratory Capacity Decreased in Arm and Leg Muscle in Type 2 Diabetes?. Diabetologia.

[59] Hey-Mogensen M., Højlund K., Vind B.F., Wang L., Dela F., Beck-Nielsen H., Fernström M., Sahlin K. (2010). Effect of Physical Training on Mitochondrial Respiration and Reactive Oxygen Species Release in Skeletal Muscle in Patients with Obesity and Type 2 Diabetes. Diabetologia.

[60] Hansen M., Lund M.T., Gregers E., Kraunsøe R., Van Hall G., Helge J.W., Dela F. (2015). Adipose Tissue Mitochondrial Respiration and Lipolysis before and after a Weight Loss by Diet and RYGB. Obesity.

[61] Camera D.M., Anderson M.J., Hawley J.A., Carey A.L. (2010). Short-Term Endurance Training Does Not Alter the Oxidative Capacity of Human Subcutaneous Adipose Tissue. Eur. J. Appl. Physiol..

[62] Otero-Díaz B., Rodríguez-Flores M., Sánchez-Muñoz V., Monraz-Preciado F., Ordoñez-Ortega S., Becerril-Elias V., Baay-Guzmán G., Obando-Monge R., García-García E., Palacios-González B. (2018). Exercise Induces White Adipose Tissue Browning Across the Weight Spectrum in Humans. Front. Physiol..

[63] Meinild Lundby A.K., Jacobs R.A., Gehrig S., de Leur J., Hauser M., Bonne T.C., Flück D., Dandanell S., Kirk N., Kaech A. (2018). Exercise Training Increases Skeletal Muscle Mitochondrial Volume Density by Enlargement of Existing Mitochondria and Not de Novo Biogenesis. Acta Physiol..

[64] Savikj M., Zierath J.R. (2020). Train like an Athlete: Applying Exercise Interventions to Manage Type 2 Diabetes. Diabetologia.

[65] Holten M.K., Zacho M., Gaster M., Juel C., Wojtaszewski J.F.P., Dela F. (2004). Strength Training Increases Insulin-Mediated Glucose Uptake, GLUT4 Content, and Insulin Signaling in Skeletal Muscle in Patients with Type 2 Diabetes. Diabetes.

[66] Pesta D.H., Goncalves R.L.S., Madiraju A.K., Strasser B., Sparks L.M. (2017). Resistance Training to Improve Type 2 Diabetes: Working toward a Prescription for the Future. Nutr. Metab..

[67] Abdul-Ghani M.A., DeFronzo R.A. (2009). Plasma Glucose Concentration and Prediction of Future Risk of Type 2 Diabetes. Diabetes Care.

[68] Chait A., den Hartigh L.J. (2020). Adipose Tissue Distribution, Inflammation and Its Metabolic Consequences, Including Diabetes and Cardiovascular Disease. Front. Cardiovasc. Med..

[69] Gelfi C., Vasso M., Cerretelli P. (2011). Diversity of Human Skeletal Muscle in Health and Disease: Contribution of Proteomics. J. Proteom..

