# Supplementary material for: The Response of Mitochondrial Respiration and Quantity in Skeletal Muscle and Adipose Tissue to Exercise in Humans with Prediabetes
Source: Cells. 2021 Nov 4;10(11):3013. doi: 10.3390/cells10113013 (PMC8616473; doi:10.3390/cells10113013)
Supplement: Supplementary file 1 [file cells-10-03013-s001.zip › cells-1404941-supplementary.pdf]

Supplementary Tables

Table S1. Differences between studied groups before the initiation and after completion of the exercise intervention.

NG - subjects with normal fasting glucose and normal glucose tolerance; IFG - subjects with impaired fasting glucose and normal glucose tolerance; IFG+IGT – subjects with impaired fasting glucose and impaired glucose tolerance; BMI – body mass index; HbA1c - Haemoglobin A1c; VAT – visceral adipose tissue; TG – triglycerides; HDL - high-density lipoprotein cholesterol; LDL - low-density lipoprotein cholesterol; VO2max - maximal oxygen consumption; HOMA-IR - homeostatic model assessment for insulin resistance was calculated; HOMA-β - homeostatic model assessment of beta cell function. Presented are p-values of post-hoc tests. Bold and Italic values denote statistical significance at the p < 0.05 level.

	**NG vs. IFG before**	**NG vs. IFG after**	**NG vs. IFG+IGT before**	**NG vs. IFG+IGT after**	**IFG vs. IFG+IGT before**	**IFG vs. IFG+IGT after**
**Weight (kg)**	0.937	0.986	0.836	0.961	0.992	0.999
**BMI (kg/m2)**	0.518	0.700	***0.040***	0.108	0.377	0.517
**HbA1c (%)**	0.383	0.568	***0.026***	***0.049***	0.378	0.408
**Glucose 0 (mg/dl)**	***<0.001***	0.464	***<0.001***	0.073	***0.045***	0.594
**Glucose 120′ (mg/dl)**	0.728	0.952	***<0.001***	0.684	***<0.001***	0.247
**Insulin 0' (μU/mL)**	0.365	0.973	***<0.001***	0.076	***0.001***	0.177
**Insulin 120′ (μU/mL)**	0.114	0.996	***<0.001***	***0.014***	***0.002***	***0.024***
**VAT mass (kg)**	0.861	0.993	***0.037***	0.216	0.165	0.338
**Total Chol. (mg/dl)**	1.000	0.993	0.966	1.000	0.971	1.000
**TG (mg/dl)**	1.000	0.905	0.320	0.612	0.340	0.939
**HDL (mg/dl)**	0.691	0.495	0.995	0.956	0.994	0.998
**LDL (mg/dl)**	0.952	0.984	0.991	0.988	1.000	1.000
**VO2max (ml/kg/min)**	0.326	0.999	0.283	1.000	0.977	0.996
**HOMA-IR**	***0.028***	0.877	***<0.001***	***0.017***	***0.015***	0.078
**HOMA-b (%)**	0.997	1.000	0.528	0.732	0.289	0.704
**Fat mass (kg)**	0.780	0.981	0.356	0.765	0.854	0.945
**Lean mass (kg)**	0.999	0.999	1.000	1.000	0.995	0.990

Table S2. MUSCLE mass-specific and mitochondria-specific respiratory capacity parameters in patients with Normoglycemia (NG), isolated Impaired Fasting Glucose (IFG) and Impaired Fasting Glucose and Impaired Glucose Tolerance (IFG + IGT), before and after 3 months of exercise intervention.

Presented are mean and standard error values. Bold and Italic values denote statistical significance at the p < 0.05 level.

C*I*P - complex I - linked OXPHOS capacity; C*I*+*II*P - maximal complex I + II -linked OXPHOS capacity; ETSmax - maximal capacity of the electron transfer system; Km - Km for succinate titration; C*I*P nor. - complex I - linked OXPHOS capacity normalized per citrate synthase (CS) activity; C*I*+*II*P nor. - maximal complex I + II -linked OXPHOS capacity normalized per citrate synthase activity; ETSmax nor. - maximal capacity of the electron transfer system normalized per citrate synthase activity; Km nor. - Km for succinate titration normalized per citrate synthase activity.

**Parameter (unit)**	**Normoglycemia *n* = 19**		**IFG *n* = 27**		**IFG + IGT *n* = 8**		***p*-Value**			***Post-hoc p-values***		
	**Before**	**After 3 m.**	**Before**	**After 3 m.**	**Before**	**After 3 m.**	**Baseline**	**Time**	**Time × Diagnosis**	**NG before vs. after 3m.**	**IFG before vs. after 3m.**	**IFG+IGT before vs. after 3m.**
**C*I*P (pmol mg-1 s-1)**	18.19 (±2.32)	17.87 (±1.45)	19.22 (±1.48)	18.76 (±1.91)	21.99 (±1.23)	15.46 (±1.77)	0.085	0.845	0.093	0.721	0.883	0.078
**C*I*+*II*P (pmol mg-1 s-1)**	62.98 (±4.95)	78.85 (±5.68)	59.64 (±2.59)	71.08 (±4.54)	67.36 (±5.44)	70.81 (±7.20)	0.612	0.094	0.642	0.117	0.291	0.271
**ETSmax (pmol mg-1 s-1)**	84.94 (±6.49)	97.89 (±6.95)	79.99 (±3.42)	93.22 (±5.68)	90.14 (±7.88)	91.90 (±10.34)	0.785	0.121	0.823	0.233	0.310	0.277
**Km (mM/L)**	4.00 (±0.65)	3.44 (±0.56)	3.21 (±0.55)	3.22 (±0.51)	5.28 (±0.95)	6.68 (±1.20)	0.053	0.643	0.201	0.815	0.724	0.115
**C*I*P nor. (pmol mg-1 s-1 /CS activity)**	0.42 (±0.05)	0.39 (±0.07)	0.57 (±0.06)	0.53 (±0.09)	0.78 (±0.05)	0.51 (±0.04)	0.104	0.964	0.152	0.772	0.907	0.069
**C*I*+*II*P nor. (pmol mg-1 s-1 /CS activity)**	1.73 (±0.22)	2.08 (±0.20)	1.97 (±0.09)	2.25 (±0.22)	2.47 (±0.34)	2.60 (±0.20)	0.628	0.152	0.883	0.180	0.437	0.347

**ETSmax nor. (pmol mg-1 s-1 /CS activity)**	2.60 (±0.21)	2.93 (±0.28)	2.70 (±0.06)	3.09 (±0.29)	3.30 (±0.46)	3.38 (±0.30)	0.834	0.376	0.923	0.331	0.526	0.295
**Km nor. (mM/L /CS activity)**	0.10 (±0.02)	0.09 (±0.03)	0.11 (±0.04)	0.11 (±0.03)	0.21 (±0.05)	0.27 (±0.03)	0.058	0.749	0.421	0.988	0.988	0.831

Table S3. ADIPOSE TISSUE mass-specific and mitochondria-specific respiratory capacity parameters in patients with Normoglycemia (NG), isolated Impaired Fasting Glucose (IFG) and Impaired Fasting Glucose and Impaired Glucose Tolerance (IFG + IGT), before and after 3 months of exercise intervention.
Presented are mean and standard error values. Bold and Italic values denote statistical significance at the $p < 0.05$ level.
C*I*P - complex I - linked OXPHOS capacity; C*I+II*P - maximal complex I + II -linked OXPHOS capacity; ETSmax - maximal capacity of the electron transfer system; Km - Km for succinate titration; C*I*P nor. - complex I - linked OXPHOS capacity normalized per citrate synthase (CS) activity; C*I+II*P nor. - maximal complex I + II -linked OXPHOS capacity normalized per citrate synthase activity; ETSmax nor. - maximal capacity of the electron transfer system normalized per citrate synthase activity; Km nor. - Km for succinate titration normalized per citrate synthase activity.

**Parameter (unit)**	**Normoglycemia *n* = 19**		**IFG *n* = 27**		**IFG + IGT *n* = 8**		***p*-Value**			***Post-hoc p-values***		
	**Before**	**After 3 m.**	**Before**	**After 3 m.**	**Before**	**After 3 m.**	**Baseline**	**Time**	**Time × Diagnosis**	**NG before vs. after 3m.**	**IFG before vs. after 3m.**	**IFG+IGT before vs. after 3m.**
**C*I*P (pmol mg-1 s-1)**	0.38 (±0.04)	0.67 (±0.18)	0.45 (±0.04)	0.64 (±0.12)	0.40 (±0.20)	0.43 (±0.01)	0.428	***0.030***	0.079	***0.011***	***0.021***	***0.040***
**C*I+II*P (pmol mg-1 s-1)**	0.93 (±0.09)	1.60 (±0.45)	1.18 (±0.06)	1.33 (±0.23)	0.94 (±0.20)	1.12 (±0.04)	0.137	***0.039***	0.257	***0.004***	***0.014***	***0.041***
**ETSmax (pmol mg-1 s-1)**	1.12 (±0.10)	1.97 (±0.54)	1.29 (±0.10)	1.59 (±0.24)	1.15 (±0.15)	1.65 (±0.01)	0.201	***0.009***	0.303	***0.004***	***0.013***	***0.008***

**Km (mM/L)**	1.81 (±0.41)	2.35 (±0.09)	1.66 (±0.51)	3.87 (±1.75)	2.65 (±1.23)	2.91 (±1.58)	0.752	0.097	0.674	0.782	0.080	0.29
**C*I*P nor. (pmol mg-1 s-1 /CS activity)**	0.26 (±0.03)	0.48 (±0.06)	0.32 (±0.03)	0.49 (±0.11)	0.30 (±0.03)	0.36 (±0.02)	0.539	***0.042***	0.081	***0.018***	***0.029***	***0.042***
**C*I+II*P nor. (pmol mg-1 s-1 /CS activity)**	0.65 (±0.06)	1.28 (±0.08)	0.84 (±0.04)	1.02 (±0.21)	0.90 (±0.08)	0.95 (±0.01)	0.482	***0.045***	0.287	***0.007***	***0.012***	***0.039***
**ETSmax nor. (pmol mg-1 s-1 /CS activity)**	0.78 (±0.07)	1.36 (±0.10)	1.01 (±0.07)	1.28 (±0.21)	1.13 (±0.09)	1.35 (±0.11)	0.740	***0.017***	0.521	***0.005***	***0.031***	***0.016***
**Km nor. (mM/L /CS activity)**	1.33 (±0.33)	1.40 (±0.45)	1.16 (±0.33)	2.36 (±1.50)	1.90 (±0.88)	2.15 (±1.87)	0.813	0.103	0.731	0.871	0.098	0.18

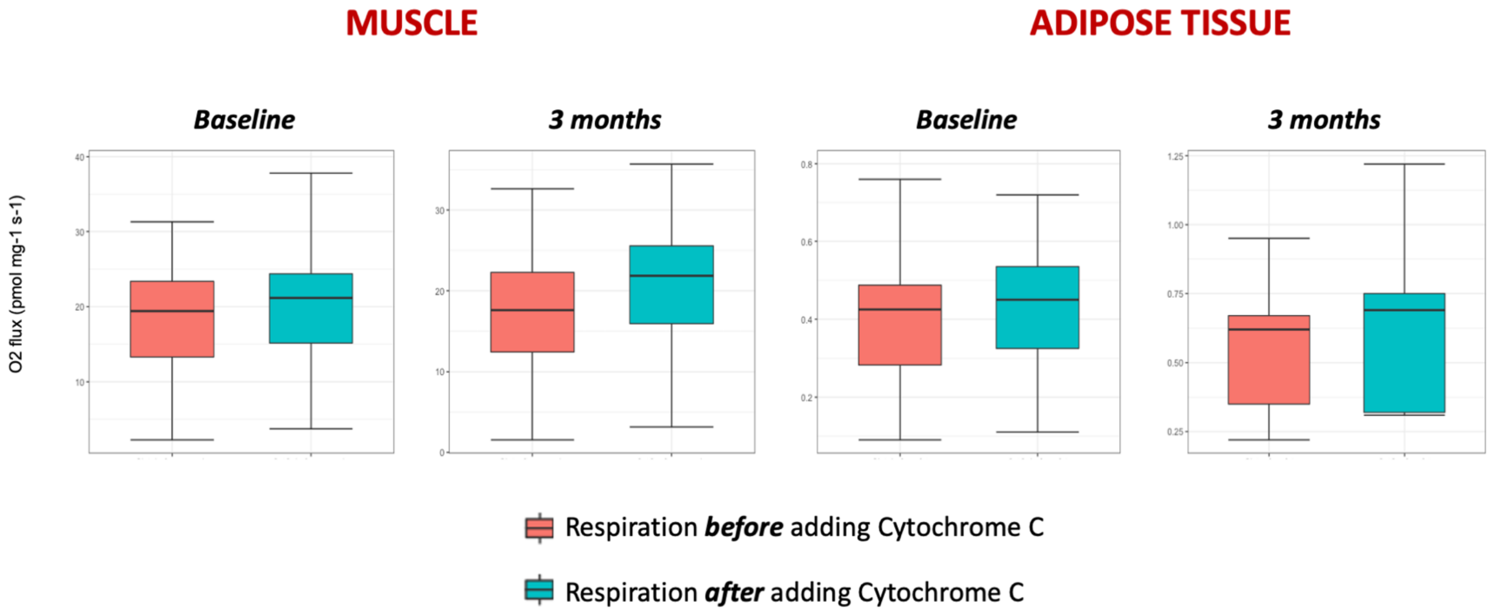

Figure S1. Changes in mitochondrial respiration after addition of Cytochrome C in adipose tissue and skeletal muscle. No significant differences were found when comparing respiration before and after addition of Cytochrome C, both before and after the exercise intervention, in adipose tissue and skeletal muscle, indicating that the integrity of the membrane was intact.
